# Reducing underreporting of stigmatized pregnancy outcomes: results from a mixed-methods study of self-managed abortion in Texas using the list-experiment method

**DOI:** 10.1186/s12905-019-0812-4

**Published:** 2019-09-03

**Authors:** Heidi Moseson, Sofia Filippa, Sarah E. Baum, Caitlin Gerdts, Daniel Grossman

**Affiliations:** 1 Ibis Reproductive Health, 1736 Franklin Street, Oakland, California 94612 USA; 2 Texas Policy Evaluation Project, Population Research Center, University of Texas at Austin, 305 E. 23rd Street Stop G1800, Austin, TX 78712 USA; 3 Advancing New Standards in Reproductive Health (ANSIRH), Bixby Center for Global Reproductive Health, Department of Obstetrics, Gynecology, and Reproductive Sciences, University of California, San Francisco, 1330 Broadway, Suite 1100, Oakland, California, 94612 USA

**Keywords:** Abortion, List experiment, Measurement error, Stigma, Survey methodology, Texas

## Abstract

**Background:**

Accurately measuring stigmatized experiences is a challenge across reproductive health research. In this study, we tested a novel method – the list experiment – that aims to reduce underreporting of sensitive events by asking participants to report *how many* of a list of experiences they have had, not which ones. We applied the list experiment to measure “self-managed abortion” - any attempt by a person to end a pregnancy on one’s own, outside of a clinical setting – a phenomenon that may be underreported in surveys due to a desire to avoid judgement.

**Methods:**

We administered a double list experiment on self-managed abortion to a Texas-wide representative sample of 790 women of reproductive age in 2015. Participants were asked how many of a list of health experiences they had experienced; self-managed abortion was randomly added as an item to half of the lists. A difference in the average number of items reported by participants between lists with and without self-managed abortion provided a population level estimate of self-managed abortion. In 2017, we conducted cognitive interviews with women of reproductive age in four states to understand how women (1) interpreted the list experiment question format, and (2) interpreted the list item on prior experiences attempting to self-manage an abortion.

**Results:**

Results from this list experiment estimated that 8% of women of reproductive age in Texas have ever self-managed an abortion. This number was higher than expected, thus, the researchers conducted cognitive interviews to better understand how people interpreted the list experiment on self-managed abortion. Some women interpreted “on your own” to mean “without the knowledge of friends or family”, as opposed to “without medical assistance”, as intended.

**Conclusion:**

The list experiment may have reduced under-reporting of self-managed abortion; however, the specific phrasing of the list item may also have unintentionally increased reporting of abortion experiences not considered “self-managed.” High participation in and comprehension of the list experiment, however, suggests that this method is worthy of further exploration as tool for measuring stigmatized experiences.

**Electronic supplementary material:**

The online version of this article (10.1186/s12905-019-0812-4) contains supplementary material, which is available to authorized users.

## Background

Self-managed abortion encompasses any attempt by a person to end a pregnancy on one’s own, outside of a clinic setting [1]. There is growing recognition that self-management of abortion is an option that people consider, and some may even prefer, for terminating unwanted pregnancies. Within the United States, Google searches related to self-management of abortion rose from 119,000 in 2011 to 700,000 in 2015 alone [2]. Two years later, more than 200,000 searches related to “self-abortion” were conducted in just one 32-day period in 2017 [3]. Estimates from a 2015 statewide representative survey in Texas suggest that approximately 1.7% of individuals of reproductive age who identify as female in have attempted to self-manage an abortion at some point in their lives [4].

Current measures of the prevalence of self-managed abortion, however, are almost certainly limited by underreporting due to legal and privacy concerns, as well as stigma [5–7]. We know this to be true for measures of abortion in clinical settings. Numerous studies have documented a tendency for participants to under-report personal experiences of abortion when asked directly in surveys, sometimes dramatically [7–10]. In one study in the United States, over 70% of participants with a history of abortion in their medical record did not disclose this abortion in a survey [6]. Fear of judgement or of others finding out may lead many individuals to choose not to disclose an abortion in a survey. For self-managed abortion in particular, fear of legal prosecution may be particularly salient as numerous women have been arrested or prosecuted for allegations of self-managed abortion in the United States [11].

Given these factors, researchers have attempted to assess the extent of underreporting of abortion through use of alternative measures (other than direct questioning), including use of the ‘best friend’ method [12], whereby respondents are asked to report on the number of abortions had by their close confidantes, rather than themselves,, as well as a method more recently introduced for abortion research: the list experiment [13–15]. The list experiment method originated in the field of social psychology in the 1980s to estimate the population proportion that holds a sensitive belief or has had a stigmatized experience [16, 17]. The method has been used frequently in the disciplines of political science and economics to measure population levels of stigmatized topics such as racism, bribery, illicit drug use, and more [18–20] – and thus, it seemed promising as a candidate method for estimating abortion, self-managed or otherwise. Indeed, the list experiment method has now been used to measure induced abortion in a handful of countries [13, 15, 21], with varied results [14].

Using the list experiment to indirectly measure abortion asks respondents to report *how many* of a list of health experiences they have experienced, one of which is abortion. The respondent does not report which specific events, just a number. Through careful selection of control items on the list to include experiences with expected (ideally documented) prevalence in the target population, analysis of these numeric responses should enable the researcher to estimate the population proportion that has experienced abortion [22]. As an individual respondent does not have to provide a definitive ‘yes’ or ‘no’ to the specific experience of abortion, the respondent may feel less at risk and more comfortable including an experience of abortion in their tally of personal experiences, thereby reducing underreporting.

Under the hypothesis that current estimates of the number of people who have attempted to self-manage an abortion likely underestimate the true number, we set out to pilot the list experiment method to generate a more complete estimate of the prevalence of self-managed abortion in Texas, and additionally, through cognitive interviews, to understand participant comprehension of the list experiment itself. We hypothesized that the list experiment would generate an estimate of self-managed abortion higher than those returned by direct questioning, and that phrasing of list items could alter participant interpretation of the self-managed abortion item.

## Methods

### List experiment study population and survey administration

For the quantitative survey in which the list experiment was administered, the GfK Group (Gesellschaft für Konsumforschung, “Society for Consumer Research”, the market research firm that conducted the survey, formerly Knowledge Networks) sampled households from its nationally representative KnowledgePanel with probability proportional to size based on key geo-demographic dimensions to create a population representative sample for the state of Texas. Participants were selected for inclusion in the national KnowledgePanel via probability-based sampling of addresses from the United States Postal Service’s Delivery Sequence File (DSF) [23]. Household members from randomly sampled addresses from the DSF were invited to join the sample through a series of mailings, and follow-up telephone calls (where addresses could be matched to a corresponding landline). Households without Internet connection were provided with a web-enabled device and free Internet service to participate in surveys. To be eligible for participation in this particular survey, a panel member must have been between the ages of 18–49 years, a resident of the state of Texas, non-institutionalized, and self-identified as female. Participants provided informed consent before beginning the survey, and were awarded a point system incentive (that translates to several US dollars) for survey completion.

Survey questions asked about reproductive history, experiences seeking sexual and reproductive health care, and sociodemographic characteristics. In addition to the list experiment question (described below), the survey also asked about experiences attempting to end an unwanted pregnancy on one’s own, without medical assistance, via a direct question, as well as by asking participants if their best friend had ever attempted to do so. The list experiment question was asked first in the survey, while the direct and best friend questions were asked later in the survey after a definition of self-managed abortion was provided. Results from these questions are presented elsewhere [4]. Post-stratification design weights accounted for non-response and any under- or over-coverage imposed by the design. Members were invited to participate in this survey between December 2014 and January 2015.

### List experiment question

**Fig. 1:**
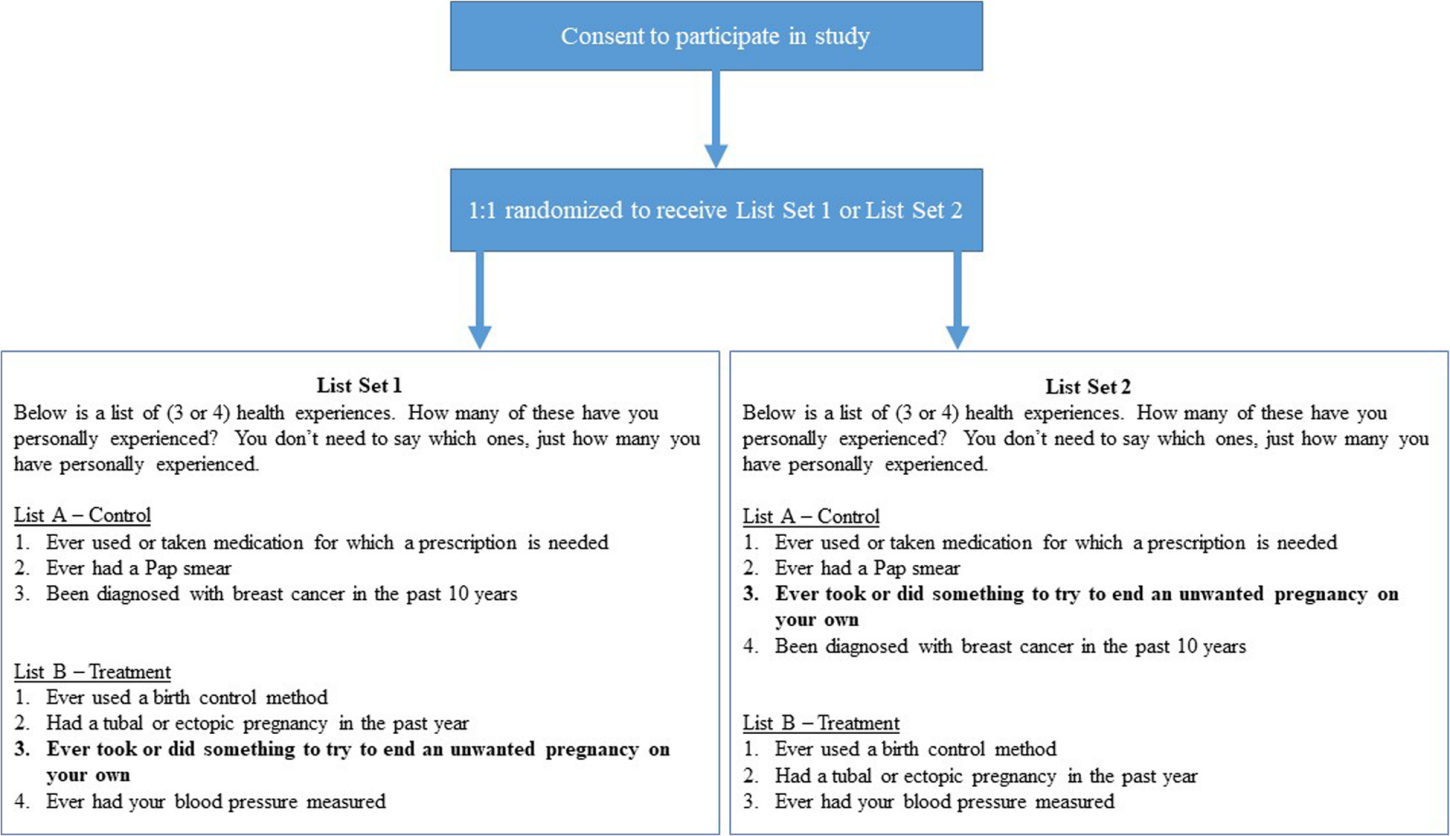
Administration of the double list experiment

For the list experiment, all respondents received two lists of reproductive health related events or experiences. Using a random number generator coded into the survey, half of the sample received List set 1, and the other half of the sample received List set 2 (Fig. 1). In this sense, the two groups served as a control for the other, each receiving one list with only non-sensitive items, and the other list with the self-managed abortion item added. Participants were asked to report *how many* of the list items were true for them, not which ones. Control list items were selected based on known frequencies of these events in the Texas population. The sensitive item phrasing for self-managed abortion read: “Ever took or did something to try to end an unwanted pregnancy on your own”. Investigators hoped that this phrasing would prompt respondents to report attempts to end a confirmed pregnancy that took place outside of a clinic setting, without help from a clinician, and to exclude attempts to *prevent* an unwanted pregnancy, such as taking Plan B or contraception in general (as these do not constitute ending a pregnancy, as a pregnancy has not yet occurred).

### List experiment analysis

A difference in means calculation between the average counts of events reported for both lists (with and without the self-managed abortion item) was then generated. These two difference-in-means estimates, one from the two versions of List 1 and one from the two versions of List 2, were then averaged to provide a more precise estimate of the population proportion of individuals that has ever attempted to interrupt a pregnancy on their own. Thus, the lifetime cumulative incidence of attempting to end an unwanted pregnancy on one’s own in this sample can be estimated using the average of two difference-in-means calculations, one for List 1 and one for List 2:

$$ \uppi =1/{\mathrm{N}}_1\ {\Sigma \mathrm{Y}}_{\mathrm{T}=1,\mathrm{i}}-1/{\mathrm{N}}_0\ {\Sigma \mathrm{Y}}_{\mathrm{T}=0,\mathrm{i}} $$

Where π represents the proportion of the population that has attempted to end an unwanted pregnancy as estimated by a single list (List A or List B), T represents which version of the list the individual received (treatment or control), N_1_ is equal to the number of individuals who received the treatment version of a given list, and N_0_ is equal to the number that received the control version of that list. The variance for individual list estimates is calculated using the standard large-sample formula for difference-in-means. The 95% confidence interval for the combined list estimate is a more tailored calculation, estimated using the variance of the control and treatment versions of each list, as well as their covariance [22]. Estimates are presented with and without the post-stratification weights created by GfK. Data analyses were conducted in Stata version 15 and R (https://www.R-project.org).

### Cognitive interview study population

In 2017, cognitive interview participants were identified at community- and clinic-based sites in four states: Alabama (Birmingham), California (San Francisco), Indiana (Bloomington), and Texas (Dallas, El Paso, and the Lower Rio Grande Valley). Clinic sites included abortion clinics, general reproductive health clinics, and HIV treatment centers. Community sites included public parks, a coffee shop, a community college, and Craigslist. Sites were selected across the United States to recruit individuals with a broad range of reproductive experiences, including individuals known to have self-managed abortion experience. Participants were selected from multiple states, beyond Texas, to inform the use of the list experiment method in upcoming surveys on self-managed abortion to be administered to more geographically diverse populations. Individuals who self-identified as female between the ages of 18–49 years who spoke English or Spanish were eligible to participate. A primary recruiter and interviewer was identified for each site to invite potential interview subjects to participate in the study, to screen for eligibility, to review informed consent materials, obtain verbal consent, and conduct the interviews. Interviews were conducted in person or by phone in the participant’s language of choice (English or Spanish).

### Cognitive interview content

The objective of the cognitive interviews was to better understand how participants (1) interpreted the list experiment question format, and (2) interpreted the list item asking about prior experiences attempting to self-manage an abortion (“Ever took or did something to try to end an unwanted pregnancy on your own”). Cognitive interview questions prompted participants to reflect on their subjective interpretations of survey questions related to self-management of abortion, with particular emphasis on the list experiment format. Specifically, participants were read four individual variants of list item questions asking about experience with self-induction and asked to describe what each meant to her, what she thought the question was trying to ask, what self- induction methods came to mind, how she interpreted specific phrases, suggestions for improving the clarity of question text and format, and more. The four wording options presented were as follows: (1) Ever took or did something to try to end an unwanted pregnancy on your own; (2) Ever took or did something to try to end an unwanted pregnancy on your own, without medical assistance; (3) Ever taken anything on your own to try to bring back your period or end a pregnancy; and (4) Ever taken or done anything on your own to try to self-induce an abortion. Cognitive interview questions also assessed participant’s thoughts on the list experiment format itself, including probes to ascertain why the participant believed the list experiment question was structured as it was, what it was trying to measure, the clarity of list experiment instructions, understanding of individual list items, and confidence in their response. The full guide can be found in the Additional file 1. Each participant received a $25 gift card for her time.

### Cognitive interview analysis

All interviews were audio-recorded and professionally transcribed. The full research team agreed on a preliminary codebook based on questions included in the cognitive interview guide, and then two researchers independently applied this codebook to the same two transcripts. After joint review and comparison of the two parallel-coded transcripts by the two researchers, the codebook was revised to accommodate more specific guidelines on code application, and to include new themes identified in the transcripts. The revised codebook was subsequently applied to all transcripts to organize content across thematic areas using the online software Dedoose.

## Results

### List experiment survey sample

Nearly all survey participants (760, or 98%) responded to the list experiment questions. Of the 760 respondents that completed the list experiment, 37% were younger than 30 years, 44% identified as Hispanic, 12% as non-Hispanic Black, and 36% as non-Hispanic White (Table 1). Twelve percent of subjects disclosed a prior abortion, more than half had attended at least some college (60%), and 22% of participants completed the survey in Spanish.

**Table 1:** Characteristics of 760 individuals who completed the list experiment in the quantitative, state-wide survey

	(%)^a^
Age, years	
18–29	37
30–39	48
40–49	15
Race/ethnicity	
Black, non-Hispanic	12
White, non-Hispanic	36
Hispanic	44
Other, non-Hispanic	7
Marital Status	
Married	50
Living with partner	12
Not married	38
Educational attainment	
High school or less	39
Some college	34
College degree	26
Language of survey	
English	78
Spanish	22
Prior induced abortion	12
Prior self-managed abortion attempt (direct question)	1.7

^a^weighted percentages

### List experiment results

We found no evidence for a design effect in either list set (List set A: *p* = 0.99; List set B: *p* = 0.94). The weighted results estimate that 8.6% (95% CI: 4–14%) of the population had ever attempted to end an unwanted pregnancy on their own. When restricted to individuals who reported ever having had intercourse with a man, the list experiment estimated that 8.2% (95%CI: 3–13%) had ever attempted to end an unwanted pregnancy on their own (Table 2).

**Table 2:** List experiment estimates of abortion self-induction attempts. All numbers are percentages

	List 1 Estimate	List 2 Estimate	Final List Estimate	95% CI
Unweighted Results				
Overall	13.9	6.0	9.9	4–15
Ever had sex	15.2	7.7	11.5	5–17
Weighted Results				
Overall	11.8	5.5	8.6	4–14
Ever had sex	10.7	5.6	8.2	3–13

### Cognitive interview sample

Twenty-six individuals participated in the cognitive interview portion of the study: four in Birmingham, Alabama, four from the Dallas, Texas area, four from El Paso, Texas, six in the Lower Rio Grande Valley of Texas, four in Bloomington, Indiana, and four in San Francisco, California. On average, participants were 26 years old (range: 20–44 years), 13 identified as Hispanic, six as non-Hispanic White, and four as non-Hispanic Black, 10 disclosed a prior abortion, and 5 disclosed a prior attempt to end an unwanted pregnancy on their own (Table 3). Of the five reported prior experiences with self-managed abortion, one was not known to the research team at the time of recruitment.

**Table 3:** Characteristics of 26 cognitive interview participants

	n
Age, years	
18–29	18
30–39	6
40–49	2
Race/ethnicity	
Black, non-Hispanic	4
White, non-Hispanic	6
Hispanic	13
Other, non-Hispanic	3
Marital Status	
Married	7
Living with partner	6
Not married	13
Educational attainment	
High school or less	8
Some college	9
College degree	9
Language of interview	
English	19
Spanish	7
Prior induced abortion	10
Prior self-managed abortion attempt (direct question)	5

### List item phrasing

All participants were asked to provide interpretations of four variants of the list experiment item asking about experience with self-management of abortion. One of the variants, “Have you ever taken or done something to try and end an unwanted pregnancy on your own?”, was the text used in the list experiment fielded in Texas in 2015. The most common interpretation of this text was having an abortion outside of a clinic setting, without medical assistance or supervision (*n* = 11/26).

*“To me, it means have I done something, like, outside of a doctor’s office or in a health setting myself at home to try to end an unwanted pregnancy (…) Without the, you know, without the benefit of a health care provider. That’s what that means to me.” -Indiana, age 35–39*

Four other women mentioned self-induction of abortion on their own, but without explicitly mentioning the lack of medical involvement. Other interpretations included having an abortion secretively or without the support or knowledge of friends, partners or family members, regardless of location (*n* = 4/26):

*"On my own" to me means literally on my own, like independently, in private, probably, by myself." – Texas, age 20-24.*

For others, this item could include an in-clinic abortion where pills were dispensed at the clinic and the abortion completes at home.

*“Yeah. I think of at-home abortions when I read that. And then "taken," I think of, don't you have - don't they have, some medicine that you can take? Even, like, the doctor can give it to you and send you home with it and it'll, like, make you have an abortion. So, that would be on my own, too, because I didn't do it at the hospital.” – Alabama, age 35–39*

Similarly, others might include an in-clinic abortion under this item so long as the person made the decision to have the abortion independently, or paid for the procedure without any help (*n* = 3/26).

*"Like, on my own, my own decision--not necessarily with your money or something like that" – Texas, age 25–29, Spanish speaker*

Of the four item variants, the most preferred phrasing read: “Have you ever taken or done something to try and end an unwanted pregnancy on your own, without medical assistance?” The main difference between this question and the prior phrasing was that adding “without medical assistance” seemed to change the abortion experiences that could be included in this category. For instance, a number of participants felt that this language allowed the respondent to include abortions done with social support from peers, partners, or family; whereas, in the previous question, these abortions were excluded because they were not thought to be done truly “on one’s own”.

*“The previous question with that aspect of on your own was a little unclear as to whether it meant truly alone in doing these things to yourself or having somebody there to help you who just may not me a medical professional, but may still be knowledgeable about what they are doing or they are ready to help you (…) For example, if this had been my experience I would be more likely to explain a situation where a friend had helped me do something like this than I would have in the other one because it wouldn’t have truly been on my own.” – Indiana, age 20–24*

Few respondents preferred the other two phrasing options tested in the cognitive interviews (“Ever taken anything on your own to try to bring back your period or end a pregnancy”; and “Ever taken or done anything on your own to try to self-induce an abortion”). Many participants commented that “bring back your period” was too vague and that it did not resonate with the language they used to talk about abortion. One participant captured this viewpoint as follows:

*“Bring back your period or end a pregnancy? That seems like two very different questions. […]”*

*“I think I understand what it's trying to get at, which might be that using "bring back your period" as another way to say end a pregnancy or not be pregnant? I mean, I think I feel like that's what you're trying to ask. But I think that there are other contexts that "bring back your period" would work in. And, I don't think anyone uses that terminology. I've never, ever heard, you know, hey, have you seen my - have you heard about Jessica? She brought back her period. It's, you know, she had a miscarriage. She had an abortion. She, you know, took the day after pill.” – Alabama, age 35–39*

While a number of participants appreciated the clarity and precision of the “self-induce an abortion” language, they also felt that the word “abortion” might carry too much stigma and “scare people away” and thus cause respondents to under-report abortion experiences as a result.

### Methods of self-managed abortion

For each of the four phrasing options, participants were asked about the methods of abortion that the wording brought to mind. Participants provided varied responses, including abortion pills, contraceptives, Plan B, tea, herbs, home remedies, and other methods such as falling down the stairs, hitting oneself in the stomach, or abusing alcohol or other substances. When asked about “on your own,” participants mentioned medications and contraceptive methods more often, as compared to when asked about “on your own without medical assistance,” when slightly more participants mentioned dangerous methods of abortion self-induction such as using a hanger, punching one’s stomach, or falling down the stairs. All methods mentioned by participants for each of the phrasing options are presented in Table 4.

**Table 4:** Self-managed abortion methods brought to mind for participants by different phrasings of the self-managed abortion list item

	Total^a^	Phrasings			
		“on your own”	“on your own without medical assistance”	“bring back your period”	“self-induce an abortion”
Drugs or alcohol	12	4	3	2	3
Teas or herbs	19	4	4	5	6
Hitting oneself/causing oneself physical harm	21	5	7	1	8
Hanger	10	3	4	1	2
Medication/pill	34	10	10	7	7
Plan B	10	3	1	4	2
Contraceptives	11	4	1	4	2
Home remedies/toxic substances	15	2	3	6	4
Suction/In clinic	7	3	0	3	1

^a^Participants often mentioned the same methods for more than one phrasing, and thus, total numbers add to more than the total sample size of *n* = 26

### List experiment format

The majority of interviewees (*n* = 20) understood the list experiment instructions, provided answers in the correct format, and felt confident in their responses. Participants who understood the list experiment questions correctly gave numbers as their answers to indicate how many of the listed experiences they had experienced, rather than specifying which items they had experienced. Despite accurate responses to the question format, not all participants understood why the question was being asked. One participant provided a succinct description of this viewpoint:

*“I feel like it’s noninvasive because someone doesn’t have to check all that apply. But I’m not sure if it gets you the answer you’re looking for. But on the responder end, I would feel comfortable with putting a number because you’re not going to be able to [know which I’ve done] – or maybe you can. But when I first think, I’m like, oh, yes, I’ll just say what it is.” – California, age 25–29*

Several respondents hypothesized that the question was structured to measure individual’s access to the listed sexual and reproductive health services, rather than any individual item:

*Interviewer: “What do you think these series of these two questions next to each other, what do you think they are trying to ask? What are they trying to understand?”*

*Respondent: “Probably access. That’s kind of my interpretation of that, is you’re probably trying to understand what kind of health care you have received and what you’ve had access to. That’s basically what I get from it.” – Indiana, age 30–34*

Most felt confident that the interviewer could not know if they had experienced any particular item on the list: *“Yeah, so it’s kind of trying to protect us from personal information too.”- Texas, age 20–24.* Some, however, felt that the interviewer might be able to tell which specific items on the list they had done, although this did not seem to deter them from answering honestly.

*Interviewer: Do you feel like I know, well, yes, she maybe had a pap smear, and yes, she's used birth control? Or, do you think there's no way for me to know which items you have done?*

*Respondent: I'm pretty sure there's a way. Between asking both questions, there has to be some sort of pattern.*

*Interviewer: Okay. So, did that - did your feeling that way make you want to change the way you answered?*

*Respondent: Oh, no. I would still answer. I would feel comfortable. – California, age 25-29*

Among participants who did not find the list experiment instructions clear, some wanted to provide a yes/no response for each list item, while others provided a numeric response, but were not confident that this was the correct way to answer the question. Beyond the format of the list experiment, we found that a number of respondents were not familiar with some of the individual list items, specifically “pap smear” (*n* = 4) and “tubal/ectopic pregnancy” (*n* = 9), and as a result, some felt that they did not know how to answer the question.

## Discussion

Using a novel method of measuring abortion experiences – the list experiment – we estimated that approximately 8% of women of reproductive age in Texas have attempted to end an unwanted pregnancy on their own at some point in their lifetime. In cognitive interviews, we found that the list experiment to measure self-management of abortion was understandable to a majority of participants, and most felt confident in their responses.

The list experiment estimate of self-managed abortion is several orders of magnitude larger than the estimate generated by a direct question about abortion self-management in this same study sample (direct question estimate: 1.7%) [24]. Similarly, another indirect measure of abortion self-management asked in the same survey found double the magnitude of the direct estimate: 4.1% of participants reported a best friend ever having attempted to end an unwanted pregnancy on her own, without medical assistance [4]. While intriguing, the difference between the direct estimate and the list estimate could reflect a number of factors other than a true exponentially larger lifetime prevalence of self-management of abortion. First, the list item measuring experience with abortion self-management was phrased differently than the direct question. The text of the list question read: “Ever took or did something to try to end an unwanted pregnancy on your own”, versus “Ever took or did something to try to end an unwanted pregnancy on your own, without medical assistance” as was used for the direct question. Thus, it is possible that some of the difference in estimates reflects the more specific language used for the direct question.

The difference between the list estimate (~ 8%) and the direct estimate (~ 2%) was larger than anticipated, and, coupled with the difference in question phrasing, prompted further investigation through cognitive interviews. We hypothesized that the presence or absence of the phrase “without medical assistance” may have resulted in participants responding differently simply because they felt that the questions referred to different experiences. Findings from the cognitive interviews suggest that participants did, in fact, interpret the phrasings to refer to different sets of self-managed abortion experiences. Some participants felt that the phrasing used in the list experiment referenced a narrower subset of abortion experiences – only those conducted on one’s own without anyone else knowing, even a partner or friend – while other participants felt that the list phrasing referred to a wider range of experiences, including in-clinic abortions as long as the individual did not tell anyone about the abortion, or in-clinic abortions as long as the decision was made on one’s own. For instance, some participants interpreted “on your own” not to mean “without medical assistance” as intended, but instead to mean “without the knowledge of my partner, friends or parents.” In addition, findings from the cognitive interviews indicate that women may have interpreted the question accurately, but incorrectly categorized the use of Plan B or other contraceptive methods as self-managed abortion (Table 4). As a result, the list experiment estimate likely overestimated the prevalence of self-managed abortion as defined by the investigators.

Another possible explanation for the difference in estimates of self-managed abortion between the two methods is that survey respondents may have felt more comfortable disclosing a prior self-managed abortion attempt via the list experiment question because of the anonymity and confidentiality that the method allows. In that case, the 8% estimate generated by the list experiment could be closer to the true lifetime prevalence of self-managed abortion attempts for women in Texas.

To understand our estimate of self-managed abortion in context, we looked to several prior studies that have estimated the lifetime prevalence of self-managed abortion among various populations in the United States. In a nationally representative survey of abortion patients seeking care at 87 abortion clinics and physicians’ offices in the United States, the percentage of abortion clients reporting ever having attempted to self-induce an abortion using misoprostol or other substances was 2.6% in 2008, and 2.2% in 2014 [25]. In a convenience sample of 1425 ever-pregnant individuals recruited from primary care, OBGYN, and abortion clinics in 2009, 4.6% reported a history of attempting self-induction [1]. Among abortion patients surveyed in Texas in 2014, 7% had taken or done something to try to end their current pregnancy before coming to the clinic [26]. Among respondents to a 2017online survey about self-abortion, 11% of 1235 participants searching for self-abortion information reported ever attempting to self-abort [3].

In all of the studies above, the samples were selected in such a way that we would reasonably expect that reported attempts to self-manage abortion would be higher than in the general population. For instance, samples of abortion patients or individuals searching for information on self-abortion are likely to differ in important ways from the general population in terms of history of unwanted pregnancy, and perhaps other characteristics related to self-managed abortion attempts. However, given that 7% of abortion patients in Texas disclosed attempting to self-abort for the current pregnancy alone – and likely, this 7% is an underestimate due to stigma and privacy concerns – it is worth considering that the true prevalence is higher than direct estimates suggest.

To our knowledge, this is both the first study to use a list experiment to measure experiences with self-managed abortion and the first study to conduct cognitive interviews about a list experiment to measure abortion. The data are limited by the fact that cognitive interviews were not conducted among respondents to the survey. Thus, we can only infer what survey respondents might have been thinking based on responses from cognitive interview participants. Additionally, ordering of the list experiment question versus the direct and best friend questions about abortion self-management may have influenced differences in response. As participants answered the list experiment without any definition provided for what it means to “end a pregnancy on one’s own”, but then answered the direct and best friend questions after reading such a definition, responses to the two direct questions may refer to a different set of abortion experiences than was referenced for the list experiment question. Cognitive interviews were designed, in part, to explore this possibility, and confirmed that interpretation did differ between phrasings.

Results from this study, however, are strengthened by the population representative sample for the quantitative survey, and by the geographic diversity of participants in the cognitive interviews – factors that may increase the generalizability of results for research in other areas of the United States. Further, this study represents a unique example of pursuing additional research to investigate surprising or unexpected research findings. The combined results from survey responses and cognitive interviews add important insight into ongoing research on self-managed abortion, including information on the ways in which individuals think about and respond to different word choices, interpretation of the list experiment format, and more.

## Conclusions

Measuring experiences of abortion self-management is necessary to understand the changing reproductive health needs and preferences of the population, to inform harm-reduction strategies if and where necessary, and to provide an indication, however imperfect, of the accessibility of family planning services. Improving our measures of self-managed abortion and tracking the prevalence of these experiences over time could also provide useful data for evaluating the impact of policies related to abortion and contraception care. More work is needed on a national level to help meet the need for safe, legal abortion care, whether in a health facility or via expansion of service delivery models to include de-medicalization. In future research, the high comprehension of the list experiment method reported by cognitive interview participants suggests that the method may be a worthwhile tool to assess self-managed abortion in the United States. Future research on this topic may do well to use the phrase “on your own without medical assistance” to more specifically capture the experience of self-managed abortion as defined by family planning researchers, with an awareness of the broad range of methods this phrasing may bring to mind for participants.

## Additional file

Additional file 1: Cognitive Interview Guide. (DOCX 33 kb)

## Data Availability

The quantitative list experiment data has been uploaded along with this manuscript. The cognitive interview instrument guides and codebook are available upon reasonable request to the corresponding author. Due to our commitment to the confidentiality of our interview subjects and obligations to our ethics review board, we are unable to provide interview transcripts beyond the excerpts included in the manuscript.

## Abbreviations

DSF: United States Postal Service’s Delivery Sequence File, from which individuals were sampled for the quantitative survey
GfK: Gesellschaft für Konsumforschung, “Society for Consumer Research”, the market research firm that conducted the survey
IRB: Institutional Review Board
