# Supplementary material for: Reducing underreporting of stigmatized pregnancy outcomes: results from a mixed-methods study of self-managed abortion in Texas using the list-experiment method
Source: BMC Womens Health. 2019 Sep 3;19:113. doi: 10.1186/s12905-019-0812-4 (PMC6720920; doi:10.1186/s12905-019-0812-4)
Supplement: Supplementary file 1 — Cognitive Interview Guide. (DOCX 33 kb) [file 12905_2019_812_MOESM1_ESM.docx]

Interview No. : _________

Date of Interview:_________

**Cognitive Interview Guide**

Thank you so much for taking the time to meet today. My name is X, and I am a researcher working with the University of Texas, Austin / Ibis Reproductive Health. The goal of this study is to better understand how women understand and interpret questions about ending unwanted pregnancies. Women make different choices about how to end an unwanted pregnancy. Some women may go to a hospital, clinic, or doctor’s office to have an abortion. Other women may do something to try to end a pregnancy without medical assistance. For example, they may get information from the internet, a friend, or family member about pills, medicine, or herbs they can take on their own, or they may do something else to try to end the pregnancy. We hope that information from this study will help us collect more accurate information about women’s abortion experiences, so that we can better design programs and policies to improve women’s health. The interview should last about 30 minutes, but if you ever feel uncomfortable with any questions, we can just move on to the next. Just let me know. If you need any question clarified, just stop and ask me. Please take your time answering any of these questions - I don’t expect you to have an answer right away. And there are no right or wrong answers, just your own thoughts and reflections. Do you have any questions now? If not, let’s begin.

**To start out, can you tell me a little bit about your background?**

- Where did you grow up?
- Where do you live now?
- Have you ever been pregnant?
- Do you have any children?

In the past, we have conducted studies asking women about their experiences with trying to end a pregnancy. Now, we are conducting these interviews (like this one right now) to better understand how women interpret some of the questions that we ask. If it’s ok, I’d like to read you some of the questions we ask and get your thoughts.

#### Here’s the first question. I am going to read one health experience that is true for some people.

#### Ever ****took or did something to try to end an unwanted pregnancy on your own?****

- Tell me what you are thinking after hearing this question.
- What does this question mean to you?
- What do you think the question is trying to ask?
- How do you interpret “on your own”?
- How do you define an unwanted pregnancy?
- How do you interpret “end a pregnancy”?
- What methods come to mind when you hear this question?
  - If she mentions Plan B, probe to understand more about why she thinks of this as self-induction, and if timing matters (whether woman takes it before or after she knows she’s pregnant)
- If we were to ask you this question about the past year only, how confident would you feel in your ability to accurately recall any such experience?
  - What about if we asked about the past 5 years? How accurate do you think your recall would be?
  - What about the past ten years?
  - Is there a certain window of time that you think is most accurate? Least likely to be mis-remembered?
- Any thoughts on how this question could be made clearer?

#### Now I’d like to read you another health experience that is true for some people, with different phrasing.

#### Ever ****took or did something to try to end an unwanted pregnancy on your own, without medical assistance?****

- **What are you thinking when you hear this question?**
- **Does it mean anything different to you than the former question?**
- **What does “without medical assistance” mean to you here?**
- **What does “on your own” here mean to you?**
- **Does this question bring to mind different methods of ending a pregnancy than the former question?**
- **Any thoughts on how this question could be made clearer?**

**Here is yet another version: Have you ever** **taken anything on your own to try to bring back your period or end a pregnancy?**

- Tell me what you are thinking after hearing this question.
- What does this question mean to you?
- Does it mean something different to you than either of the former two questions?
- What do you think the question is trying to ask?
- How do you interpret “bring back your period”?
- What methods come to mind when you hear this question?
- Any thoughts on how this question could be made clearer?

#### Thank you. What about the following question: Have you ever taken or done anything on your own to try to self-induce an abortion?

- Tell me what you are thinking after hearing this question.
- What does this question mean to you?
- How do you interpret “self-induce an abortion”?
- What methods come to mind when you hear this question?
- Any thoughts on how this question could be made clearer?

#### Thinking back on the above questions, I presented four different options to you to try to measure women’s experience with non-clinical abortion. The options were, as a reminder:

1. **Ever took or did something to try to end an unwanted pregnancy on your own**
2. **Ever took or did something to try to end an unwanted pregnancy on your own, without medical assistance**
3. **Ever taken anything on your own to try to bring back your period or end a pregnancy**
4. **Ever taken or done anything on your own to try to self-induce an abortion**

- Do you think any of the above is more or less clear than the others?
- Do you prefer one wording to the other?
- Do they all mean the same thing to you, or do they mean different things?
- Are women more or less likely to truthfully respond to one of the above items as compared to the others?
- Which do you think we should ask?

**For the above questions, we generally read them to women in the following format. Please listen to the below question and let me know what you think.**

**Below is a list of 3 health experiences. How many of these have you personally experienced?  You don’t need to say which ones, just how many you have personally experienced.**

- **Ever used or taken medication for which a prescription is needed**
- **Ever had a Pap smear**

#### ****Been diagnosed with breast cancer in the past 10 years****

#### ****Answer choices: 0, 1, 2, 3****

#### ****Below is list of 4 different health experiences. How many of these have you personally experienced?****  Again, you don’t need to say which ones, just how many.

#### ****Ever used a birth control method****

#### ****Ever took or did something to try to end an unwanted pregnancy on your own****

#### ****Had a tubal or ectopic pregnancy in the past year****

#### ****Ever had your blood pressure measured****

#### ****Answer choices: 0, 1, 2, 3, 4****

- Tell me what you are thinking after hearing these questions.
- What do you think the question is trying to ask?
- Does the format make sense to you?
- Did you feel confident in your answer?
- Why do you think the question is structured in this way?
- Do you feel that I can tell if you have done any specific item on the list?
- What does a “pap smear” mean to you?
- What does a “tubal or ectopic pregnancy” mean to you?
- Were the instructions clear? What could be done to make the instructions more clear?
- Any thoughts on how the format of these questions could be made clearer?

Ok. Thank you. Now, I would like to ask you another question and get your thoughts.

**“Has your best friend ever taken or used something on her own, without medical assistance, to try to end an unwanted pregnancy? The answer choices are Yes, No, I suspect she did this, and I don’t know.”**

- Tell me what you are thinking after hearing this question.
- What does this question mean to you?
- What do you think the question is trying to ask?
- How do you interpret “best friend”?
  - Upon hearing this question, if you yourself had an experience ending a pregnancy on your own but didn’t want to admit to it, would you respond to this question with information about yourself, under the guise that it was about your “best friend”? In other words, would you feel more comfortable disclosing your experience if the interviewer thought it was about someone else, not yourself?
  - Do you think women respond to this question about themselves, as opposed to their “best friends”? Why or why not?
- What do you think of the answer choices? Are there options missing?
- How do you interpret the answer choice “I suspect she did this”?
- Any thoughts on how this question could be made clearer?

Another variant of some of the questions we ask is sometimes worded as follows:

**“Have you ever taken anything on your own to try to bring back your period or end a pregnancy? (check all that apply”**

**Yes, I have taken cytotec, or misprostol**

**Yes, I have taken emergency contraception, also known as EC or the morning-after pill**

**Yes, I have taken another drug: ________**

**None of the above”**

- Tell me what you are thinking after hearing this question.
- What does this question mean to you?
- What do you think the question is trying to ask?
- What does “emergency contraception” or “EC” mean to you?
- What does the “morning after pill” mean to you?
- Do you have other words for these pills?
- What do you think of when you hear these words?
- Any thoughts on how this question could be made clearer?

Thank you so much for your responses. I have another question to read to you related to a medication.

**“What have you heard about Cytotec, misoprostol, “the star pill”, or the pill to "bring down your period”?”**

- Tell me what you are thinking after hearing this question.
- What does this question mean to you?
- What do you think the question is trying to ask?
- Have you heard the word Cytotec before? Misoprostol? The star pill? The pill to bring down your period?
- In what context?
- What do you think of when you hear these words?
- Any thoughts on how this question could be made clearer?

Related to the above questions, we often ask women about a list of specific methods that some women use to try to end a pregnancy without clinical supervision. These methods are listed as follows:

- **Misoprostol or Cytotec**
- **Emergency Contraception (the “morning after pill” or Plan B) before confirming she was pregnant**
- **Emergency Contraception (the “morning after pill” or Plan B) after she knew she was pregnant**
- **Some other drug or medication**
- **Herbs**
- **She hit herself in the abdomen**
- **Something else**
- Tell me what you are thinking after hearing the above options.
- Are there any options that you are unfamiliar with?
- Are there any options that you know of that are not listed?
- Any way that this list could be made clearer or more complete?

Ok great. I have one last question for you, somewhat related to the above, and this time I’m just interested in your opinion – not how you interpret the question. And to reiterate, there are no right or wrong answers. We are interested in how women think about and form opinions on these issues, not on whether something is factually correct or not.

**In your experience, either for yourself, for your friends, or based on other information, why do you think women take Plan B?**

- Probe to understand whether she think women take Plan B to END a pregnancy, versus taking Plan B to PREVENT a pregnancy.
- Follow-up question: If a woman is worried she might get pregnant and takes Plan B to prevent a pregnancy, how would you describe what she did?
  - Do you think this could be considered self-induction of abortion?
- What if a woman knows that she is pregnant (or strongly thinks she might be), and takes Plan B in an attempt to end the pregnancy? How would you describe what she did?
  - Do you think this could be considered self-induction of abortion?

Thank you so much. Just to wrap up, we’d love to ask you a few basic questions that we can use to better describe women’s experiences more generally. As always, feel free to not answer any question.

- How old are you now?
- What is the highest level of education you have completed?
- Do you identify as belonging to any religious faith?
- With what race or ethnicity do you most identify?
- How would you describe your relationship status?
- How many children do you have?
- Have you ever had an abortion?
- Have you ever self-induced an abortion?

**Closing Script:** That is all of the questions that I have on my end. Is there anything that you would like to talk about? Do you have any questions, about what we discussed today, or the study more generally? *If no, or after addressing further questions*: I want to thank you again for taking the time to talk with me today. We are so grateful to you for your time. If you have any other questions that come up, please feel free to email or call us at any time using the contact information we provided for you.
